# Oral Antiviral Defense: Saliva- and Beverage-like Hypotonicity Dynamically Regulate Formation of Membraneless Biomolecular Condensates of Antiviral Human MxA in Oral Epithelial Cells

**DOI:** 10.3390/cells13070590

**Published:** 2024-03-28

**Authors:** Pravin B. Sehgal, Huijuan Yuan, Anthony Centone, Susan V. DiSenso-Browne

**Affiliations:** 1 Department of Cell Biology and Anatomy, New York Medical College, Valhalla, NY 10595, USA; huy20@pitt.edu; 2 Department of Medicine, New York Medical College, Valhalla, NY 10595, USA; 3 Department of Pathology, Microbiology and Immunology, New York Medical College, Valhalla, NY 10595, USA; acentone@student.touro.edu; 4 Touro College of Dental Medicine at New York Medical College, Hawthorne, NY 10532, USA; susan_disenso@touro.edu

**Keywords:** barrier immunity, oral/gingival epithelium, environmental hypotonic stress, interferon-λ/IL-29, human myxovirus resistance protein (MxA), biomolecular condensates, membraneless organelles (MLOs), osmoregulation, macromolecular crowding, regulated volume decrease (RVD)

## Abstract

The oral mucosa represents a defensive barrier between the external environment and the rest of the body. Oral mucosal cells are constantly bathed in hypotonic saliva (normally one-third tonicity compared to plasma) and are repeatedly exposed to environmental stresses of tonicity, temperature, and pH by the drinks we imbibe (e.g., hypotonic: water, tea, and coffee; hypertonic: assorted fruit juices, and red wines). In the mouth, the broad-spectrum antiviral mediator MxA (a dynamin-family large GTPase) is constitutively expressed in healthy periodontal tissues and induced by Type III interferons (e.g., IFN-λ1/IL-29). Endogenously induced human MxA and exogenously expressed human GFP-MxA formed membraneless biomolecular condensates in the cytoplasm of oral carcinoma cells (OECM1 cell line). These condensates likely represent storage granules in equilibrium with antivirally active dispersed MxA. Remarkably, cytoplasmic MxA condensates were exquisitely sensitive sensors of hypotonicity—the condensates in oral epithelium disassembled within 1–2 min of exposure of cells to saliva-like one-third hypotonicity, and spontaneously reassembled in the next 4–7 min. Water, tea, and coffee enhanced this disassembly. Fluorescence changes in OECM1 cells preloaded with calcein-AM (a reporter of cytosolic “macromolecular crowding”) confirmed that this process involved macromolecular uncrowding and subsequent recrowding secondary to changes in cell volume. However, hypertonicity had little effect on MxA condensates. The spontaneous reassembly of GFP-MxA condensates in oral epithelial cells, even under continuous saliva-like hypotonicity, was slowed by the protein-phosphatase-inhibitor cyclosporin A (CsA) and by the K-channel-blocker tetraethylammonium chloride (TEA); this is suggestive of the involvement of the volume-sensitive WNK kinase-protein phosphatase (PTP)-K-Cl cotransporter (KCC) pathway in the regulated volume decrease (RVD) during condensate reassembly in oral cells. The present study identifies a novel subcellular consequence of hypotonic stress in oral epithelial cells, in terms of the rapid and dynamic changes in the structure of one class of phase-separated biomolecular condensates in the cytoplasm—the antiviral MxA condensates. More generally, the data raise the possibility that hypotonicity-driven stresses likely affect other intracellular functions involving liquid–liquid phase separation (LLPS) in cells of the oral mucosa.

## 1. Introduction

The oral mucosa represents a defense boundary between the external environment and the rest of the body and encompasses several antimicrobial and antiviral mechanisms [1,2,3,4,5,6]. Oral mucosal cells are constantly bathed in hypotonic saliva (normally one-third tonicity compared to plasma; isotonicty is approximately 300–330 mOsm), and are also repeatedly exposed to the environmental stresses of tonicity, temperature, and pH by the drinks we imbibe (e.g., hypotonic: water, tea, and coffee; hypertonic: assorted fruit juices and red wines), as well as of diverse microbial and viral flora [3,4,5,6]. In the mouth, the broad-spectrum human antiviral mediator MxA (“myxovirus resistance factor A”) is constitutively expressed in healthy gingival epithelium and is also induced by Type III interferons (IFNs) such as IFN-λ1. MxA structures are also observed in the cytoplasm of oral epithelial cells in inflammatory lesions [7,8]). MxA is a 60-kDa dynamin-family large GTPase induced in many cell types by exposure to Type I (IFNs-α and β) or III (IFN-λ) interferons but not Type II interferon (IFN-γ) [9,10,11,12,13,14]. Remarkably, only IFN-λ (a Type III IFN) and not IFN-α (a Type I IFN) induce MxA in oral epithelial cells [1] (also see below). The Type III IFNs comprising the IFN-λ species are the primary “barrier” IFNs at the level of the oral cavity [1]. To clarify the nomenclature, by convention, the human gene is called *Mx1* and the human protein is called MxA; however, some investigators also refer to the protein as Mx1 [11,12,13,14].

Human MxA is exclusively cytoplasmic and inhibits the replication of a broad range of RNA- and DNA-containing viruses including influenza, vesicular stomatitis virus (VSV), SARS-CoV-2, herpes, papilloma, and adenoviruses [9,10,11,12,13,14]. The molecular mechanisms by which human MxA inhibits cytoplasmically replicating RNA viruses such as VSV is primarily by inhibition of early viral transcription (within 45 min of the start of the infection) [15,16,17,18]; soluble (dispersed) MxA inhibits VSV-virion-driven transcription in cell-free assays [17], and it has been suggested that it is the free dispersed MxA in the cell cytoplasm that is antivirally active [18]. The GTPase activity is necessary for most but not all of the antiviral activities of MxA; moreover, dimerization of cytosolic MxA is critical for antiviral activity [11,12,13,14,15,16,17,18].

Four years ago, we recognized that the granular cytoplasmic structures formed by endogenous human MxA and exogenously expressed human GFP-MxA in the cytoplasm of human liver and lung cancer cells (Huh7 and A549 respectively) represented novel liquid–liquid phase-separated (LLPS) membraneless biomolecular condensates [19,20,21,22,23,24]. These “membraneless organelles” (MLOs) were devoid of an enveloping membrane as evaluated by correlated light and electron microscopy (CLEM), had a gel-like internal milieu as judged by fluorescence recovery after photobleaching (FRAP), and disassembled rapidly (in 1–2 min) when cells were exposed to 1,6-hexanediol (5% Hex), a hydrophopic reagent which disrupts phase-separated condensates [19,20,21,22,23,24]. Importantly, MxA condensates required an intact cellular plasma membrane to maintain their integrity in the cytoplasm [19]. Indeed, cytoplasmic MxA condensates in liver and lung cancer cells turned out to be sensitive sensors of hypotonicity—the condensates disassembled within 1–2 min of exposure of cells to hypotonicity (range 40–100 mOsm), and reassembled in 1–2 min but into new condensate structures when cells were returned to isotonic medium (300–330 mOsm) [19,20,24]. Remarkably, there was spontaneous reassembly of MxA condensates in 5–7 min in A549 lung cancer cells even when kept continuously at one-fourth or one-third tonicity medium (range: 80–100 mOsm) [24]. Since saliva corresponds to approximately one-third isotonicty (approx. 100–110 mOsm) and we regularly imbibe hypotonic drinks such as water, tea, and coffee (range 5–40 mOsm) [4,5,25], our observations about the dynamic nature of MxA condensates raised the possibility that MxA structures, and thus their antiviral biology, in oral/gingival cells might be sensitive to the hypotonicity-driven environmental stresses that we inflict repetitively on our oral mucosa every single day. 

In the present study, we explored the dynamic cell biology of the newly discovered membraneless MxA condensates in the cytoplasm of human oral epithelial cells. We investigated the physiological regulation of MxA condensate dispersal and reassembly by saliva- and beverage-like hypotonicity in oral carcinoma cells in culture. We tested the hypothesis that “macromolecular uncrowding, and then recrowding” of cytosolic content, due to cell-volume increase, and then regulated volume decrease (RVD), determined the equilibrium between dispersed MxA and MxA in condensates in oral cells. Biochemically, the data implicate the well-known WNK kinase-protein phosphatase (PTP)-K-Cl cotransporter (KCC) pathway [26,27,28,29] as a mechanism underlying RVD in oral epithelial cells. Taken together, physiologically, hypotonic drinks (water, tea, and coffee) would trigger a rapid deployment of antiviral MxA throughout the cytoplasm of oral epithelial cells, with subsequent spontaneous salvage of MxA back into storage granules/condensates for further cycles of antiviral defense.

## 2. Materials and Methods

### 2.1. Cells and Cell Culture

Human oral carcinoma cell line OECM1 was purchased from Millipore-Sigma (St. Louis, MO, USA). Human lung adenocarcinoma cell line A549 was obtained from the ATCC (Manassas, VA, USA). Additional aliquots of the A549 cells and its derivative line A549-hACE2 were obtained from BEI Resources/ATCC (Manassas, VA, USA). The respective cell lines were grown in DMEM (Corning Cat. No. 10-013-CV, with glutamine, Na-pyruvate and high glucose) supplemented with 10% *v*/*v* fetal bovine serum (FBS; Gibco, Grand Island, NY, USA) in T25 flasks [19,22,24]. For experiments, the cells were typically plated in 35 mm dishes without or with cover-slip bottoms [19,22,24]. Recombinant human IFN-α2 was purchased from BioVision (Milpitas, CA, USA) and recombinant human IFN-λ1 (also called IL-29) from R & D Systems (Minneapolis, MN, USA). Cells in 35 mm cultures were typically treated with these IFNs used at 50 ng/mL for 2 days. Whole-cell extracts and Western blot analyses for MxA were carried out as previously reported [19,22,24]. 

### 2.2. Plasmids and Transient Transfection

The GFP (1-248)-tagged full-length human MxA was a gift from Dr. Jovan Pavlovic (University of Zurich, Zurich, Switzerland) [17]. Transient transfections were carried out using just subconfluent cultures in 35 mm plates using DNA in the range of 0.3–2 µg/culture and the Polyfect reagent (Qiagen, Germantown, MD, USA) and the manufacturer’s protocol (with 10 µL Polyfect reagent per 35 mm plate) [19]. 

### 2.3. Live-Cell Fluorescence Imaging

Live-cell imaging of GFP-MxA structures in transiently transfected cells was carried out in cells grown in 35 mm plates using the upright the Zeiss AxioImager 2 equipped with a warm (37 °C) stage and a 40× water immersion objective, and also by placing a coverslip on the sheet of live cells and imaging using the 100× oil objective (as above) with data capture in a time-lapse or z-stack mode (using Axiovision 4.8.1 software) [19,22,24].

### 2.4. Phase Transition Experiments

Live GFP-MxA expressing cells in 35 mm plates were imaged using a 40× water-immersion objective 2–3 days after transient transfection in growth medium or serum-free DMEM medium or in phosphate-buffered saline (PBS) using methods outlined earlier [19,22,24]. Briefly, after collecting baseline images of MxA condensates, the cultures were exposed to 1,6-hexanediol (5% *w*/*v*) in PBS, or to hypotonic or hypertonic buffer of indicated strength (ELB; 10 mM NaCl, 10 mM Tris, pH 7.4, 3 mM MgCl_2_; or culture medium diluted 1:3 or 1:4 with sterile water; or culture medium adjusted to 3× tonicity with D-sorbitol) and live-cell imaging continued [24]. After the appropriate indicated times (range 5–30 min) the cultures were switched to isotonic phosphate-buffered saline (PBS) or full culture medium and imaged for another 10–15 min. Evaluation of the effects of particular inhibitors on MxA condensate disassembly and reassembly was carried out by first exposing cultures to the relevant inhibitor for 20 min in isotonic medium (full-culture medium or PBS) and then shifting to the hypotonic condition in the continued presence of the inhibitor at the original concentration.

### 2.5. Immunofluorescence Imaging

Typically, the cultures were fixed using cold paraformaldehyde (4%; PBA) for 1 h and then permeabilized using a buffer containing digitonin or saponin (50 µg/mL) and sucrose (0.3 M) [19,22,24]. Single-label and double-label immunofluorescence assays were carried out using antibodies as indicated, with the double-label assays performed sequentially. Fluorescence was imaged as previously reported [19,22,24] using an erect Zeiss AxioImager M2 motorized microscopy system with Zeiss W N-Achroplan 40×/NA0.75 water immersion or Zeiss EC Plan-Neofluor 100×/NA1.3 oil objectives equipped with a high-resolution RGB HRc AxioCam camera and AxioVision 4.8.1 software in a 1388 × 1040 pixel high-speed color-capture mode. 

### 2.6. Quantitation of Relative Amounts of GFP-MxA in Condensates vs. Dispersed State in a Cell

The procedure used to quantitate the relative amounts of MxA in the condensed vs. dispersed state on a per-cell basis as explained in Figure 2 in [20] and in Figure 7 in [24]. Briefly, images with mixed condensate and dispersed MxA were subjected to Fourier filter processing to subtract objects of small radii (2–4 pixels) in Image J. The pixel radius (in the range 2–4 pixels) used for the subtraction was optimized to subtract all condensates from the image. MxA intensity in the residual subtracted image corresponded to the dispersed protein; subtracting this from the total intensity per cell gave the % of MxA in condensates on a per-cell basis [20,24].

### 2.7. VSV Stock and Virus Infection

A stock of the wild-type Orsay strain of VSV (titer: 9 × 10^8^ pfu/mL) was a gift of Dr. Douglas S. Lyles (Department of Biochemistry, Wake Forest School of Medicine, Winston-Salem, NC, USA). Single-cycle virus infection studies at high multiplicity (moi > 10 pfu/mL) were carried out essentially as described by Carey et al. [30] as summarized in Davis et al. [19] and in Sehgal et al. [22,24]. Briefly, cultures (approx. 2 × 10^5^ cells per 35 mm plate), previously transfected with the pGFP-MxA expression vector (2 days earlier), were replenished with 0.25 mL serum-free Eagle’s medium and 10–20 µL of the concentrated VSV stock added (corresponding to MOI > 10 pfu/cell). The plates were momentarily rocked every 15 min for the first 60 min followed by addition of 0.75 mL of full culture medium. For the experiments shown in Figure 2, the cultures were fixed either at 5–6 h or 20–24 h after the start of the infection using 4% PFA in isotonic PBS (1 h at 4 °C), and immunostained for VSV nucleocapsid (N) protein using an mAb provided by Dr. Douglas S. Lyles (mAb 10G4). N-protein immunofluorescence (in red) in GFP-positive (green) and negative cells, with DAPI in blue using multicolor fluorescence imaging [19,22,29]. 

### 2.8. Antibody Reagents and Chemicals

Rabbit pAb to human MxA (H-285) (ab-95926) was purchased from Abcam Inc. (Cambridge, MA, USA); Mouse mAb to the VSV nucleocapsid (N) designated 10G4 was a gift from Dr. Douglas S. Lyles (Wake Forest School of Medicine, NC, USA). Rabbit mAb to glyceraldehyde-3-phosphate dehydrogenase (GAPDH; 14C10; number 2118) was obtained from Cell Signaling (Danvers, MA, USA). Respective AlexaFluor 488- and AlexaFluor 594-tagged secondary donkey antibodies to rabbit (A-11008 and A-11012) or mouse (A-21202 and A-21203) IgG were from Invitrogen Molecular Probes (Eugene, OR, USA). 

Cyclosporin A (CsA) was purchased from APExBIO (Houston, TX, USA); calcien-AM, calyculin-A, okadaic acid, and tetraethylammonium chloride (TEA) (a “nonselective” K-channel blocker [31]) were purchased from Millipore-Sigma (St. Louis, MO, USA).

Lipton’s black tea, pre-ground Colombian coffee, and Propel were purchased from ShopRite supermarket (Elmsford, NY, USA). Tea was brewed by soaking one tea bag in one cup of boiling water (8 oz) for 2 min; coffee was brewed in a drip percolator (2 tablespoons coffee grounds per one cup of water). Drinking water was used from a water fountain in the laboratory. All beverages were equilibrated at 37 °C before use in cell culture experiments.

### 2.9. Osmolarity Measurements

The osmolarity of various cell culture media, buffers, and beverages (100 µL aliquots) was measured using a freezing-point depression osmometer (MicroOsmometer Model 5004, Precision Systems, Natick, MA, USA). 

### 2.10. Statistical Testing

This was carried out using non-parametric one-way ANOVA (Kruskal-Wallis) with Dunn’s post-hoc test for multiple comparisons. The software used was GraphPad Prism 10.

## 3. Results

### 3.1. IFN-λ1 (IL-29), but Not IFN-α2, Induces Expression of MxA to Form Endogenous Cytoplasmic Condensates in Oral Epithelial Cells (OECM1 Cell Line)

**Figure 1:**
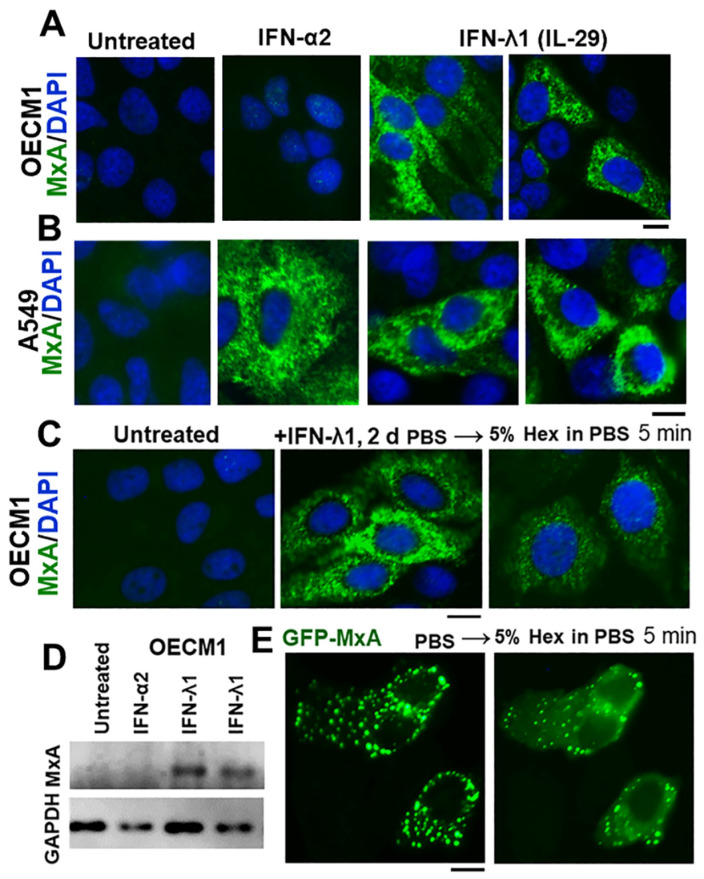
IFN-λ1 (IL-29), but not IFN-α2, induces expression of MxA in oral epithelial cells (OECM1 cell line). (**A**,**B**) 35 mm cultures of OECM1 and A549 cells were left untreated or treated with human IFN-α2 or human IFN-λ1 (50 ng/mL for 2 days) followed by fixation (4% PFA for 1 h at 4 °C) and immunofluorescence imaging for MxA; (**C**) OECM1 cultures treated with IFN-λ1 for 2 days were exposed to 5% 1,6-hexanediol in PBS, or left untreated, and then fixed and imaged for MxA; (**D**) Western blot of extracts (30 µg/lane) prepared from parallel plates as in (**A**); (**E**) OECM1 cultures were transfected with pGFP-MxA expression vector and imaged in PBS 2 days later, followed by treatment with 5%-Hex and imaging of the same cells 5 min later. All scale bars = 10 µm.

It has been previously demonstrated that MxA expression is markedly enhanced when various human cell types are exposed to Type I IFNs (i.e., IFN-α species or IFN-β) or Type III IFNs (i.e., IFN-λ species) but not by Type II IFN (i.e., IFN-γ) [9,10,11,12,13,14]. Thus, typically, IFN-α2 and IFN-λ1 both lead to MxA enhancement in human cells (see Figure 1B for A549 lung adenocarcinoma cells). However, with the growing realization that IFN-λ is the major IFN at play at the boundary between the external environment and the body, IFN-λ species have been implicated in enhancing expression of MxA in gingival cells [1,7]. It is the Type III interferons comprising IFN-λ species that primarily effectuate the immune-barrier function in the oral cavity [1] and citations therein. Thus, in order to set the baseline, we compared MxA expression in OECM1 oral carcinoma cells (“OECM1”) with that in A549 lung adenocarcinoma cells in the same experiment using aliquots from the same stock of IFN-α2 and IFN-λ1 stocks. The immunofluorescence imaging data in Figure 1A compared to that in Figure 1B show that, while both Type I and Type III IFNs enhanced expression of MxA in A549 cells (as expected), only Type III IFN (IFN-λ1) induced MxA in OECM1 oral cells. Thus, the oral epithelial carcinoma cell line OECM1 preserves the selectivity of primary gingival keratinocytes to MxA-induction primarily by Type III but not so much by Type I IFNs [1]. Evaluation of MxA protein expression in OECM1 cell extracts by Western blot assay confirmed >10-fold stimulation of expression by IFN-λ1 with little if any increase in response to IFN-α2 in these cells (Figure 1D). With this physiological selectivity (responsiveness to Type III but not to Type I IFN) established (Figure 1A,D), we used OECM1 cells for the remainder of the experiments in this study. We note that IFN-λ1-induced MxA has never before been investigated for its phase-separated biomolecular condensate nature.

In both A549 and OECM1 cells, the IFN-induced endogenous MxA was present in cytoplasmic granular structures (Figure 1A,B). We have previously shown that these cytoplasmic MxA structures induced by IFN-α2 in A549 cells represent membraneless biomolecular condensates [24] which disassemble rapidly when cells are exposed to 1,6-hexanediol (Hex), a reagent which disrupts weak hydrophobic interactions and is often used to test whether structures in cells are liquid–liquid phase-separated (LLPS) condensates [20,21]. Figure 1C shows data that IFN-λ1- induced MxA cytoplasmic granules in OECM1 cells were significantly disassembled in cells exposed to 5% Hex for 5 min, providing evidence for their condensate nature. Moreover, OECM1 cells transiently transfected with an expression vector for human GFP-MxA showed the cytoplasmic accumulation of spheroidal structures which were also disassembled significantly within 5 min when cells were exposed to 5% Hex (Figure 1E). This observation, together with the hypotonicty-induced disassembly of GFP-MxA structures (see below), provided evidence that these structures in OECM1 cells were biomolecular condensates with pools of cytoplasmic GFP-MxA in dynamic equilibrium between the condensate and dispersed phases [20,24]. 

**Figure 2:**
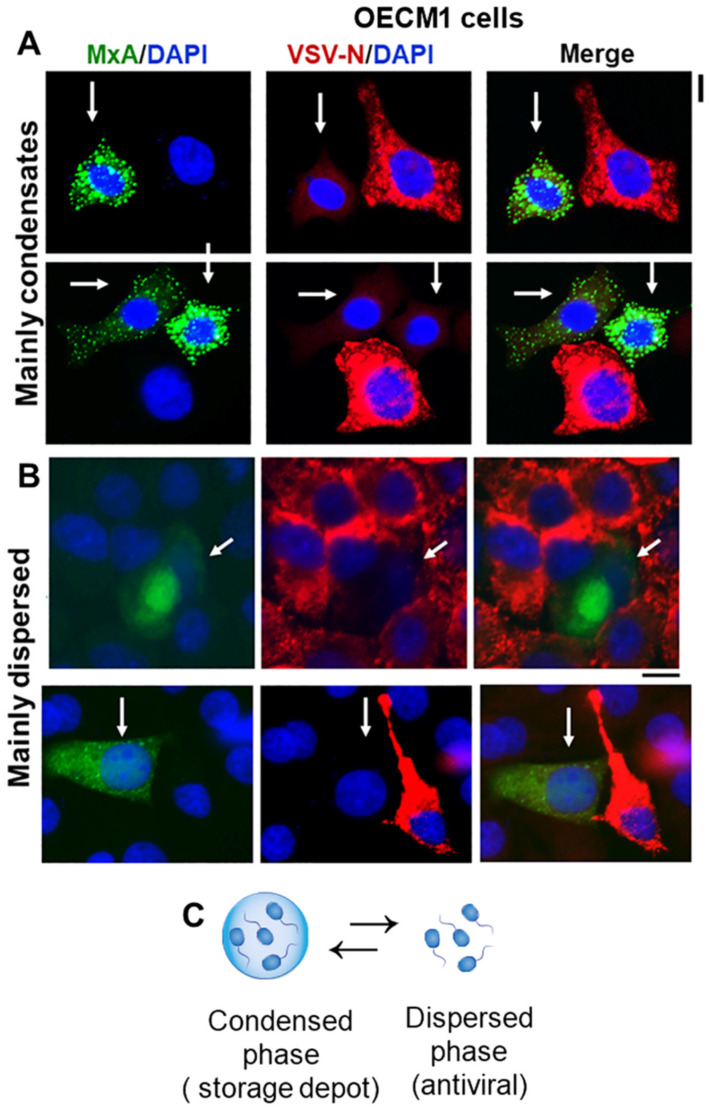
Single-cell antiviral phenotype (protected against VSV; white arrows) of OECM1 cells expressing GFP-MxA mainly in condensates (**A**) or mainly in the dispersed phase (**B**). Cultures in 35 mm plates were transiently transfected with pGFP-MxA vector, and two days later were challenged with VSV (moi > 10 pfu/cell) and fixed 24 h after the start of infection [31]. VSV replication was assessed by immunostaining for the VSV nucleocapsid (N) protein (in red) [19,24]. White arrows point to GFP-containing cells with reduced VSV-N. Scale bars = 20 µm. (**C**) Schematic highlighting the dynamic equilibrium between GFP-MxA in condensed vs. dispersed phase (note from Figure 4B, and additional Figures below, that even in cells with visually “mainly” condensed GFP-MxA, there is 15–25% of GFP-MxA in the dispersed phase, which can be still antivirally active as in (**B**) above).

Oral epithelial cells expressing GFP-MxA, either mainly in condensate form or mainly in dispersed phase, both showed an antiviral phenotype against VSV (Figure 2A,B). Since even cells with predominantly condensed GFP-MxA contain at least 5–25% of their GFP-MxA in the dispersed phase (see quantitation of various examples below), the data suggest that the condensed phase is likely a storage depot (Figure 2C) from which some or even all of the MxA can be dynamically shifted to the dispersed phase for its antiviral activity (also see Figure 5 in ref. [18] for the inference reached in 1999 from studies of MxA mutants that the cytoplasmically dispersed MxA, and not “complexes”, was the antivirally active entity). The following sections deal with a novel aspect of this dynamic bulk-shifting process triggered by hypotonicity, and physiologically regulated by parts of the volume sensitive WNK kinase-protein phosphatase-K-Cl cotransporter pathway [26,27,28,29]. 

### 3.2. Hypotonicity Sensing by GFP-MxA and Endogenous MxA Condensates

**Figure 3:**
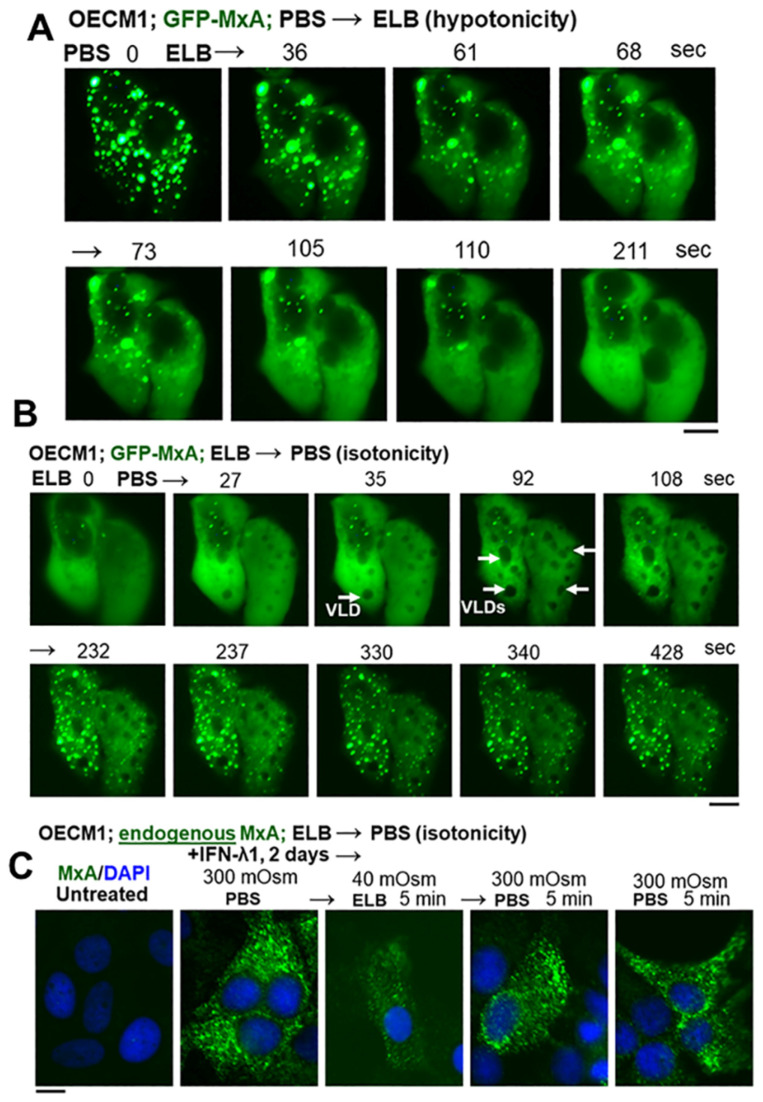
Reversible osmosensing by GFP-MxA condensates in oral epithelial cells—focus on beverage-like hypotonicity. (**A**) Sequential live-cell imaging of the same OECM1 cells expressing GFP-MxA condensates 2 days after transient transfection first in isotonic PBS (300 mOsm) and then after shifting to hypotonic ELB (40 mOsm). (**B**) the same cells as in (**A**) were sequentially imaged after further shifting back to isotonic PBS (300 mOsm). White arrows, formations of vacuole-like dilations [29] prior to condensate formation. (**C**) IFN-λ1 (50 ng/mL for 2 days) treated OECM1 cultures were fixed after washing with PBS, or after 5 min in ELB, or after 5 min in ELB, and then returned back to PBS for 5 min. Cultures were fixed using 4% PFA and immunostained for MxA. All scale bars = 10 µm.

With the realization that saliva is typically hypotonic (one-third isotonicity) and that we repetitively subject the oral mucosa to low- and high-tonicity stresses in the drinks we imbibe [5], we investigated the stability of MxA condensates in the oral epithelium to tonicity stresses. With water, tea, and coffee collectively in the range of 5–50 mOsm, we tested whether exposing OECM1 cells expressing GFP-MxA condensates and held under isotonic conditions showed any change when cells were shifted to erythrocyte lysis buffer (ELB; approx. 40 mOsm). The data in Figure 3A show that, when shifted to ELB-hypotonicity, the GFP-MxA in oral cells rapidly disassembled within 1–2 min. The data in Figure 3B show that cells with GFP-MxA in the dispersed phase rapidly reassembled into new condensates within 1–2 min of exposure of cells to isotonic PBS. These cells showed the formation of vacuole-like dilations (white arrows in Figure 3B) just prior to condensate formation. As we and others have previously pointed out [24] and citations therein, VLDs represent the internalization of “excess” plasma membrane into large cytoplasmic vesicles as swollen cells shrink back to a normal volume. Importantly, even IFN-λ1-induced *endogenous* MxA condensates showed disassembly in hypotonic ELB, and then reassembly upon shift back to isotonic PBS (Figure 3C). Overall, the data in Figure 3 recapitulate the dynamical behavior of GFP-MxA condensates previously observed in liver and lung adenocarcinoma cells in response to hypotonicity and shift back to isotonicity [19,24].

Many investigators have reported that hypertonicity can also drive proteins into cytoplasmic condensates [29]. In the case of GFP-MxA condensates in oral cells shifting cells to 3× isotonicity (achieved using D-sorbitol) to match that of assorted fruit juices or red wines [5], did not affect condensates preexisting in OECM1 cells (Figure S1). 

**Figure 4:**
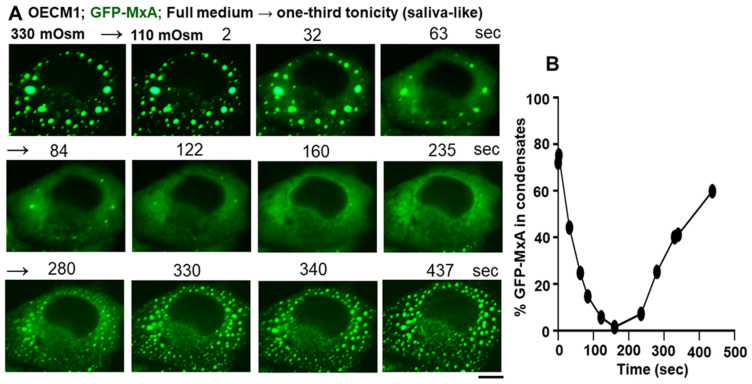
Spontaneously reversible osmosensing by GFP-MxA condensates in oral epithelial cells-focus on saliva-like hypotonicity. (**A**) Sequential live-cell imaging of the same OECM1 cell expressing GFP-MxA condensates 2 days after transient transfection first in full-culture medium (330 mOsm) and then after shifting to hypotonic of one-third tonicity (110 mOsm; full medium diluted 1:2 with water) for the next 8–10 min. Scale bar = 10 µm. (**B**) Quantitation of GFP-MxA in condensates on a % per-cell basis in the images shown in (**A**). This quantitation was carried out using the small object subtract Filter in Image J [20,24].

**Figure 5:**
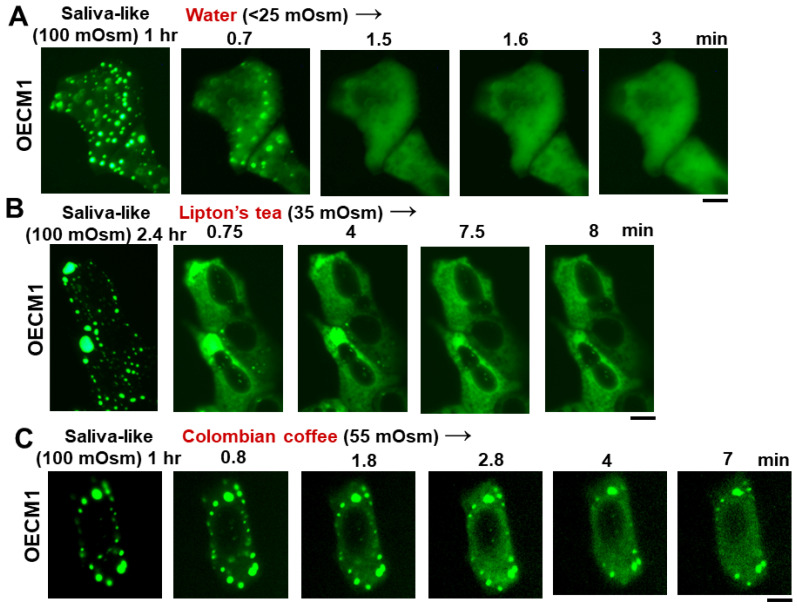
Rapid disassembly of GFP-MxA condensates by drinking water, tea, and coffee. OECM1 cultures expressing GFP-MxA condensates (2 days after transfection) were first imaged in full medium (approx. 330 mOsm) and then shifted to one-third tonicity saliva-like medium (100 mOsm) for 1–2.4 h to allow completion of the disassembly and reassembly cycle as shown in Figure 4. Single live cells in the respective cultures were then imaged and the imaging continued upon shifting the cultures to drinking water (**A**), tea (**B**), coffee (**C**). Scale bars = 10 µm.

In as much as saliva is approximately one-third isotonic (range 80–110 mOsm) [3,4,5,6], we investigated the effect of saliva-like tonicity on GFP-MxA condensates (Figure 4). OECM1 cells exhibiting GFP-MxA condensates in isotonic culture medium were shifted to one-third tonicity medium (full medium diluted 1:2 using water), and the same cell imaged for the next 10–15 min (and kept continuously in hypotonic medium). Figure 4A shows a typical cell evidencing condensate disassembly within 1–2 min of hypotonic shift. Remarkably, the condensates reappeared 5–6 min later even in cultures maintained under saliva-like hypotonic conditions. The new condensates were different from the original ones (Figure 4A). Quantitation of % GFP-MxA in condensates was carried out using Image J and a Fourier filter algorithm that subtracts objects of small radius [20,24]. This quantitation, shown in Figure 4B, revealed a rapid dynamical disassembly of condensates in 1–2 min followed by a “spontaneous” reassembly in the next 5–8 min. Following this reassembly, GFP-MxA condensates (and the oral epithelial cells) remained intact in one-third tonicity medium (100 mOsm) for up to 24 h mimicking the existence of oral epithelial cells in saliva. Figure 5 shows examples of GFP-MxA condensates in OECM1 cells 1 to 2.4 h after a shift down to 100 mOsm medium (left-most panels in each of Figure 5A–C). Figure 5 also shows what happened upon exposing such cells (starting with saliva-like conditions) to drinking water, black tea (unsweetened, no milk), or black coffee (unsweetened, no milk), which are all hypotonic. In each instance, the GFP-MxA condensates rapidly disassembled within minutes while the cells remained intact. The data in Figure S2 show that GFP-MxA condensates previously disassembled in cells exposed to drinking water or tea rapidly reassembled (within 2–4 min) when exposed to isotonic phosphate buffered saline or Propel (Pepsico) drink. Taken together, the data in Figure 3B,C, Figure 4, and Figure 5 suggest a biological mechanism in which saliva and hypotonic drinks lead to rapid deployment of antivirally active GFP-MxA into the cytoplasm, followed by subsequent retrieval of the MxA back into storage granules/condensates. The biophysical and biochemical mechanisms underlying this reversible process were then investigated.

### 3.3. Biophysical Mechanism: Macromolecular Uncrowding and Recrowding during Regulated Volume Changes

Cell-volume increase in response to hypotonicity involves the passive entry of extracellular water into cells through aquaporin channels. This leads to a dilution of cytosolic content denoted as macromolecular uncrowding. Additionally, this volume increase activates the WNK kinases (WNK1-4), which in turn activate a reverse recovery process called regulated volume decrease (RVD). RVD includes the active exit of potassium and chloride ions through KCC co-transporter channels (KCC1-4) in a charge-neutral manner; this leads to cell-volume decrease through the accompanying exit of water [26,27,28,29]. RVD results in an increase in macromolecular concentration/crowding in the cytosol [32,33]. Calcein-AM is a nonfluorescent ester which readily permeates the plasma membrane [32,33]. In the cell, cleavage of the ester releases free membrane-impermeable calcein, which is fluorescent. However, this fluorescence intensity is quenched by cytosolic compounds and macromoleculaes, and is thus an excellent reporter for the status of “macromolecular crowding” in the cytosol.

We hypothesized that the biophysical basis for GFP-condensate disassembly in oral cells exposed to hypotonic liquids was cell swelling and resulting macromolecular “uncrowding” of the cytosol. And, in reverse, condensate reassembly in cells returned to isotonic medium, or during spontaneous recovery, was due to macromolecular “recrowding” of the cytosol. This hypothesis was tested using calcein-AM preloading (to generate the intracellular calcein reporter) in an experiment parallel to that in Figure 3A,B. 

**Figure 6:**
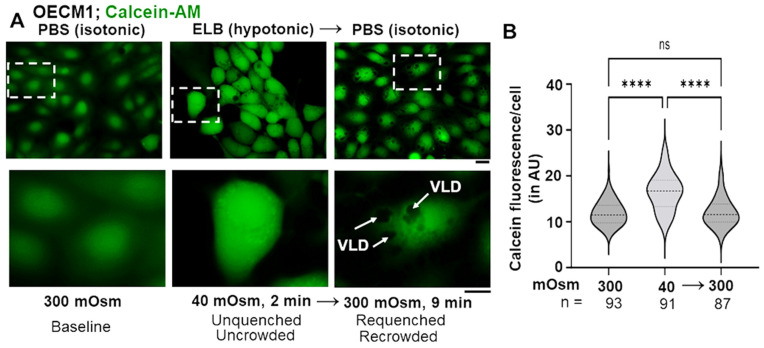
Testing a biophysical basis for hypotonicity sensing by GFP-MxA condensates in oral epithelial cells using calcein quenching as a reporter for macromolecular crowding. (**A**) OECM1 cells were preloaded with calcein-AM (2 µM in PBS) for 15 min, washed 4× with PBS and then imaged. The culture was then shifted to hypotonic ELB for 2 min and imaged immediately using the same fluorescence settings. The culture was then shifted to isotonic PBS for 9 min and cells imaged. Areas within white dashed boxes are shown at higher magnification in the lower panels. Scale bar = 10 µm. (**B**) Calcein fluorescence on a per-cell basis (in arbitrary units) is depicted. ****, *p* < 0.0001; ns, not significant; VLD, vacuole-like dilatations; n, number of cells quantitated.

Figure 6A illustrates representative fluorescence imaging of OECM1 cells first preloaded for 15 min with calcein-AM in isotonic PBS (for 15 min), followed by shifting to hypotonic ELB for 2 min, and then returned back to isotonic PBS for 9 min. Quantitation of this fluorescence is summarized in the violin plots in Figure 6B. The data provide evidence for cytosolic uncrowding and then recrowding as cells were cycled through hypotonicity and then back to isotonicity. Thus, the biophyisical basis for GFP-MxA condensate disassembly and reassembly as illustrated in Figure 3A,B, is likely cytosolic macromolecular uncrowding and then recrowding.

### 3.4. Biochemical Mechanisms: Volume-Sensitive WNK Kinase-Protein Phosphatase-K-Cl-Cotransporter Pathway

The biochemical bases for cell-volume regulation includes a pathway involving volume-change-triggered activation of the WNK kinases (of which there are four: WNK1-4), which in turn activate a reverse recovery process involving activation of protein phosphatases (of which there are three major ones: PTP1, PTP2A, PTP2B), which in turn dephosphorylate the KCC K-Cl cotransporters (of which there are four: KCC1-4) to enhance exit of K and Cl (and water) to reduce cell volume [26,29,32,33] (see Section 4 below). In reverse, hypertonicity, which decreases cell volume, drives WNK1 into cytoplasmic condensates and, through the WNK1-Spak/OSR1 pathway, phosphorylates KCC1 to decrease KCC1 co-transporter channel activity leading to cell-volume increase [29]. 

In order to test the possible involvement of this pathway in the physiological regulation of GFP-MxA condensate disassembly and reassembly, we investigated the effect of three different protein-phosphatase inhibitors (calyculin A, cyclosporin A (CsA), and okadaic acid) on GFP-MxA condensate disassembly and reassembly in oral cells under saliva-like (one-third tonicity) conditions [33,34]. Additionally, we investigated the effect of an “unselective” K-channel inhibitor (tetraethylammonium chloride, TEA) on this process [31]. In these experiments, OECM1 cells were first treated with the respective inhibitors in a full-culture medium, and then shifted to a one-third tonicity medium still containing the respective inhibitors. Live-cell imaging of the same cell(s) was carried out for 15–30 min as indicated.

**Figure 7:**
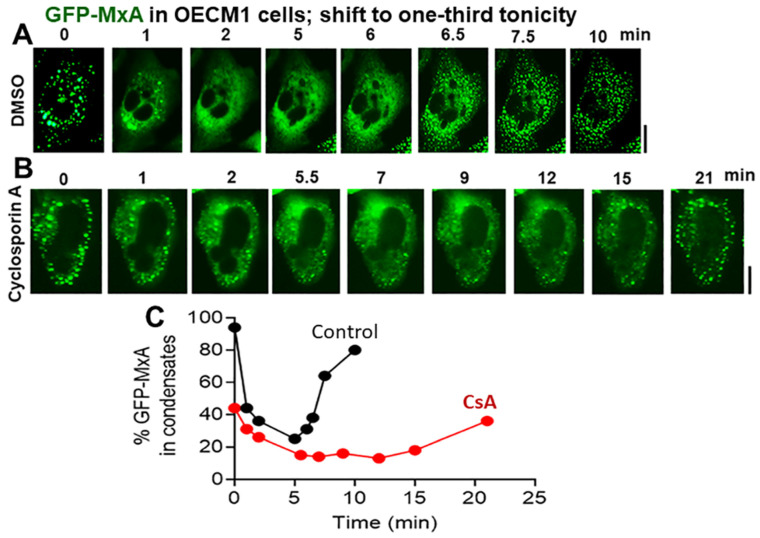
Testing a biochemical basis for hypotonicity sensing by GFP-MxA condensates in oral epithelial cells. (**A**,**B**), OECM1 cells were either exposed to CsA (25 µM) or DMSO alone for 20 min in full-culture medium, and then shifted to one-third tonicity medium in the continued presence of CsA. Live-cell imaging was carried out as indicated. Scale bar = 10 µm. (**C**) Quantitation of % GFP-MxA per cell in condensates (in the same cells shown in (**A**,**B**)).

**Figure 8:**
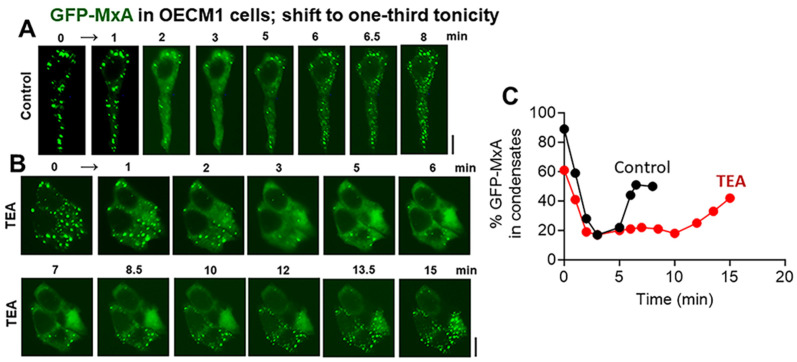
Testing a biochemical basis for hypotonicity sensing by GFP-MxA condensates in oral epithelial cells. (**A**,**B**) OECM1 cells were either kept in full-culture medium or exposed to TEA (20 mM) in full-culture medium for 20 min. Cultures were then shifted to one-third tonicity medium in the continued presence of TEA. Live-cell imaging was carried out as indicated. Scale bar = 10 µm. (**C**) Quantitation of % GFP-MxA per cell in condensates (in the same cells shown in (**A**,**B**)).

The data in Figure 7 show that cyclosporin A (25 µM; which preferentially inhibits PTP2B) [34] did not affect GFP-MxA condensate disassembly upon shift-down to one-third tonicity, but considerably slowed down condensate reassembly in oral cells. In similar experiments, the phosphatase inhibitors calyculin A (50–60 nM) and okadaic acid (1 µM) (which target PTP1, and PTP2A respectively [34]) had little effect on condensate disassembly and reassembly in oral cells. The data in Figure 8 and Figure S3 show that the K-channel inhibitor TEA (20 mM) also slowed down GFP-MxA condensate reassembly in oral cells and in A549 cells. Taken together, the data in Figure 7, Figure 8, and Figure S3 suggest the involvement of the WNK-PTP-KCC pathway in regulating GFP-MxA condensate dynamics in oral cells subjected to hypotonic stress. 

## 4. Discussion

**Figure 9:**
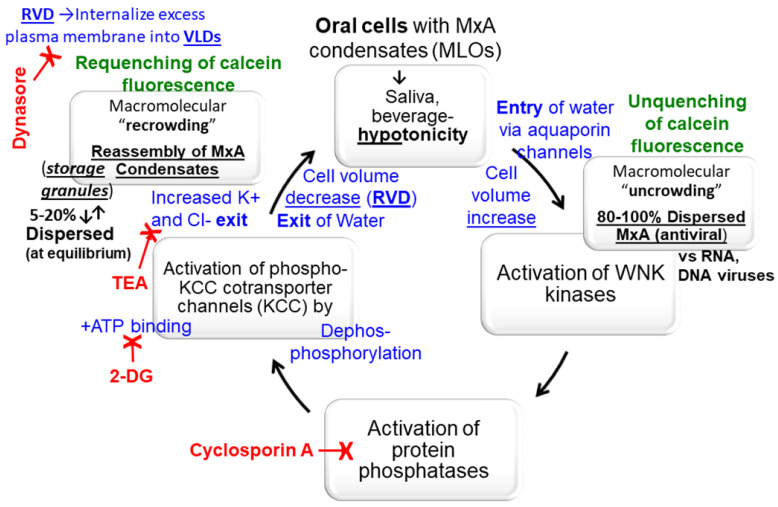
**Hypothesis:** Overview of the biophysical and biochemical mechanisms possibly involved in the cell-volume-driven dynamic regulation of the formation, disassembly, and reassembly of MxA condensates in oral epithelial cells subjected to saliva- and beverage-like hypotonicity. 2-DG, 2-deoxyglucose; KCC, potassium-chloride cotransporter channels 1–4; MLO, membraneless organelle, TEA, tetraethylammonium chloride; RVD, regulated volume decrease; VLD, vacuoele-like dilatations; WNK kinase, “With no lysine” kinase family members 1–4.

There are two aspects of novelty in the present studies. The first has to do with oral biology and oral antiviral defense mechanisms. We specifically focus on the structure and function of antiviral MxA, which is present in normal periodontal tissues [7]. Our MxA studies provide a demonstration in oral epithelial cells of the new cell biology centered on the newly recognized mechanisms of liquid–liquid phase separation (LLPS), which generate membraneless organelles (MLOs) in the intracellular space. The present studies highlight the regulation of MxA condensates in oral cells subjected to hypotonicity (Figure 9) as part of the repetitive environmental stress that we inflict on the oral mucosa when we imbibe drinks of varying tonicity (water, tea, coffee, assorted fruit juices, beers, red wines, etc.). We connect the underlying mechanisms of the regulation of macromolecular uncrowding and recrowding, likely through the WINK-PPTP-KCC pathway (Figure 9), to antiviral defense at the level of the oral cavity. Specifically, the new data highlight the role of Type III interferons such as IFN-λ1 and of MxA as an antiviral defense mechanism at the boundary between the external milieu and the oral mucosa. 

The second aspect of novelty has to do with advancing the basic science related to the cell biology and biochemistry of MxA. Briefly, our ongoing work corrects a major long-standing error in the IFN and MxA field. Since 2002, it had been widely thought that human (Hu) MxA is associated with the membrane of the smooth endoplasmic reticulum (ER) [35,36]. There is widespread adherence to this inference in the IFN and Mx fields (so quoted in hundreds of papers) [12,13]. A key basis for this is data such as those in Figure 5d from Stertz et al. [35] showing the distribution of HA-MxA following transient expression in Huh7 cells in a reticular meshwork in the cytoplasm. However, Stertz et al. [35], and earlier Accola et al. [36], admitted that none of the customary markers for the ER matched MxA structures. Yet, for two decades, the MxA field unquestioningly accepted that the meshwork shown in 2006 in data from Stertz et al. was the ER (see [35,36]). The breakthrough correction came when we discovered that Huh7 cells (and other hepatoma lines Hep3B and HepG2) contained a novel meshwork of intermediate filaments based on giantin [19,20]. HA-MxA and GFP-MxA condensates associated with this intermediate filament meshwork—this meshwork that had been misidentified by Stertz et al. as the ER [35]. Our studies move the field of MxA research into the realm of LLPS-driven biomolecular condensates [19,20,21,22,23,24]. 

Indeed, it is now increasingly recognized that LLPS leads to the formation of biomolecular condensates in the cytoplasm and nucleus of eukaryotic cells [20,21,37,38,39,40,41]. These droplets form “MLOs” and provide novel scaffolds for diverse cellular functions (e.g., cell signaling, nuclear transcription, RNA splicing and processing, mRNA storage and translation, DNA sensing, synaptic function, and mitosis) distinct from membrane-enclosed organelles [20,21,37,38,39,40,41]. It is also now recognized that the replication of many viruses in mammalian cells involves LLPS condensates (e.g., in the life cycles of VSV, rabies [Negri bodies], influenza A, Ebola, measles, Epstein-Barr, Ad5, and SARS-CoV-2 viruses [20,42,43] and citations therein).

Human MxA is a cytoplasmic 60-kDa sized dynamin-family GTPase which, in the soluble dispersed phase, associates into dimers. While the structure of MxA includes an intrinsically disordered region (IDR) called loop L4 [11,12,13,44,45], it is not clear yet whether this contributes to condensate formation. While the GTPase activity is necessary for most of the antiviral activity of MxA, this is not an absolute requirement. MxA mutated to be GTPase-null retains antiviral activity towards the hepatitis B virus [17,18,19,23]. Additionally, mutants of MxA that lack both GTPase activity and also anti-viral activity continue to form cytoplasmic condensates (and sometimes even bigger condensates compared to wild-type (wt) MxA) [11,12,13,22]. Thus, we surmise from this, and data such as in Figure 2 showing cells with predominantly dispersed MxA to retain antiviral activity (also see Figure 5 in ref. [18] for a similar inference), that the condensates likely represent a storage granule/depot from which MxA can be rapidly dispersed to effect its antiviral activity (such as inhibition of early transcription of VSV) [15,16].

The cognate-related human protein MxB is also cytoplasmic and shows antiviral activity against HIV and the herpes virus type 1 [11,12,13,17,22]. MxB, and GFP-MxB, also forms membraneless condensates on the cytosolic side of nuclear pores and thus inhibits the transit of viral RNA into the nucleus [22,46] and citations therein. The major murine ortholog of both human *MxA* and human *MxB* genes is the murine *Mx1* gene; the related protein is murine Mx1 [11,12,13,22]. Murine Mx1 has a weak nuclear localization signal; it is thus nuclear in its residence and is antiviral towards the influenza virus but not against cytoplasmic VSV [11,12,13,22]. Murine *Mx2* is not orthologous to either human *MxA* or human *MxB*; the protein is cytoplasmic in residence and is antiviral against VSV [11,12,13]. Curiously, we showed that, in many human Huh7 liver cancer cells, exogenously expressed murine GFP-Mx1 can associate with cytoplasmic intermediate filaments [22]. Such cells now show an antiviral phenotype against VSV, in contrast to those with exclusively nuclear murine GFP-Mx1 residence [22]. We recently reported that *nuclear* murine GFP-Mx1 structures were also LLPS-driven biomolecular condensates which, like cytoplasmic human MxA condensates, disassembled in cells exposed to hypotonic ELB, and reassembled in cells then shifted to isotonic PBS [22]. Thus, not only did the subcellular localization of Mx family members contribute significantly to their respective antiviral spectrum, but also the cytoplasmic human MxB and nuclear murine Mx1 formed biomolecular condensates [22,46]. For our present discussion, we note that GTPase-null mutants of human MxA, which do not have antiviral activity, continue to form cytoplasmic condensates [11,12,13,14,44,45]. With respect to human MxA, we suggest that the cytoplasmic condensates likely represent storage granules/depots of either of wt or mutant MxA species (Figure 2). The fraction of wt MxA in the cytoplasmic dispersed phase is likely the biologically antiviral mediator. Indeed, we observed that hypotonicity-driven disassembly of MxA in lung adenocarcinoma (A549) cells did not interfere with the antiviral activity of GFP-MxA against VSV [24].

Cells of the OECM1 human oral carcinoma line grow profusely in a full-culture medium under isotonic conditions. Shifting these cultures to a saliva-like one-third tonicity medium rapidly disassembled GFP-MxA condensates within 1–2 min. Maintaining these oral cells continuously in one-third tonicity led to a spontaneous recovery of GFP-MxA condensates within 4–7 min, indicative of the existence of regulatory cell-volume recovery mechanisms and, thus, the ability of OECM1 oral cells to adjust to saliva-like tonicity (Figure 9). Both condensates of exogenously expressed GFP-MxA and IFN-λ1 induced endogenous MxA showed similar disassembly and reassembly responses. Parenthetically, OECM1 cells and the GFP-MxA condensates in them survived at least 24 h under one-third tonicity conditions (Figure 5).

The biophysical mechanism for GFP-MxA condensate disassembly and its reassembly appears to be macromolecular uncrowding and recrowding in the cytosol (Figure 9). That condensate formation can be driven by macromolecular crowding both in intact cell cytoplasm and in cell-free assays is now well established [20,21,37,38,39,40,41]. The cell-activity-dependent, spontaneous, regulated “macromolecular recrowding”, and, thus, reassembly of cytoplasmic GFP-MxA condensates (Figure 9), is less understood. We obtained evidence showing the unquenching and requenching of calcein fluorescence during this process in oral epithelial cells (Figure 6). Hypotonicity is thought to drive water into cells passively through aquaporin channels as part of cell swelling and an expansion of the plasma membrane. The reverse, regulated volume decrease (RVD), involves internalization of the “excess” plasma membrane into vacuole-like dilatations (VLDs), which appears to compress cytosolic space [19,24] and, additionally, involves a biochemical pathway initiated at the volume-sensitive WNK kinases (e.g., the ubiquitous WNK1 kinase) and OSR1 kinase, leading to activation of PTPs such as PTP2B by phosphorylation [26,27,28,29]. The activated PTPs enhance activity of K-Cl cotransporter channels (e.g., the ubiquitous KCC1) through dephosphorylation. Importantly, the binding per se of ATP to KCC1 cotransporter is required for the KCC activity and the charge-neutral exit of K and Cl from the cells (accompanied passively by water) and thus a reduction in cell volume (Figure 9). 

In a previous study in A549 cells, we reported that dynasore, a dynamin inhibitor, slowed GFP-MxA condensate reassembly [24]. Additionally, pretreatment of cells to 2-deoxyglucose (2-DG), which reduces intracellular levels of ATP, also slowed spontaneous GFP-MxA reassembly in A549 cells exposed to one-third tonicity [24] (Figure 9). In oral OECM1 cells we now report (a) the appearance of VLDs during recovery from hypotonicity (Figure 3B and Figure 6A), and (b) that cyclosporin A, an inhibitor of PTP2B, and TEA, an “unselective” inhibitor of K channels, slowed reassembly of hypotonicity-dispersed GFP-MxA into condensates (Figure 7, Figure 8, and Figure S3). These data suggest that the pathways outlined in Figure 9 as the possible biochemical bases for RVD involved in the dynamic regulation of hypotonicity-driven GFP-MxA condensates merit further investigation. Moreover, the effects of environmental stressors such as temperature (hot or cold drinks) and pH (e.g., acidic drinks) on condensate biology in oral mucosal cells also merit investigation.

## 5. Limitations of This Study

The present study is limited to an investigation of GFP-MxA condensates and their dynamics using one rapidly growing human oral epithelial carcinoma cell line (OECM1). We have confirmed that, as with primary gingival epithelial cells [1], OECM1 cells display a selectivity in their response to Type I and Type III IFNs. With respect to the induction of MxA, in Figure 1, we show that an iconic Type I IFN (IFN-α2) does not induce MxA in OECM1 cells but an iconic Type III IFN (IFN-λ1) does. For now, we justify experiments using only OECM1 cells, in as much as our current focus is to set up a foundation for the detailed proteomic evaluation of GFP-MxA condensates and also the biochemistry that regulates condensate disassembly and reassembly. This requires an ample supply of bulk cells. However, we are mindful that observations derived from studies of OECM1 cells can guide us forward but will remain to be validated using primary human gingival epithelial cells.

## 6. Conclusions

The oral cavity is routinely and repetitively subjected to hypotonic stress (and hypertonic, temperature, and pH stresses). The present study identifies a novel subcellular consequence of hypotonic stress in oral epithelial cells in terms of the rapid and dynamic changes in the structure of one class of phase-separated biomolecular condensates in the cytoplasm. Specifically, the data suggest the hypotonicity-driven deployment of MxA into the cytoplasm is an enhanced antiviral mechanism, followed, eventually, by MxA retrieval back into storage granules/condensates. More generally, the data raise the possibility that tonicity-driven stresses in cells of the oral mucosa likely affect other diverse intracellular functions (signaling, transcription, translation, and innate immunity mechanisms) mediated by phase-separated biomolecular condensates. 

## Data Availability

All data are available within the manuscript and in the Supplementary Materials.

## Supplementary Materials

The following supporting information can be downloaded at: https://www.mdpi.com/article/10.3390/cells13070590/s1, Figure S1: Sorbitol hypertonicity has little effect on GFP-MxA condensates in OECM1 cells; Figure S2: Rapid reassembly of dispersed GFP-MxA into condensates triggered by isotonic phosphate-buffered saline and by Propel (Pepsico); Figure S3: TEA slows spontaneous reassembly of GFP-MxA condensates in A549 cells exposed to one-third tonicity medium.

## Abbreviations

2-DG, 2-deoxyglucose; CLEM, correlated live-cell fluorescence and electron microscopy; CsA, cyclosporin A; ELB, hypotonic erythrocyte lysis buffer; ER, endoplasmic reticulum; FRAP, fluorescence recovery after photobleaching assay; GAPDH, glyceraldehyde 3-phosphate dehydrogenase; GFP, green fluorescent protein; HA, influenza virus hemagglutin tag; Hex, 1,6-haxanediol; IDR, intrinsically disordered region; IFN, interferon; KCC, potassium-chloride cotransporter; LLPS, liquid-liquid phase separation; MLO, membraneless organelle; MxA, human myxovirus resistance protein A; post-infection; PBS, phosphate-buffered saline; PM, plasma membrane; PTP, protein phosphatase; RVD, regulated volume decrease; TEA, tetraethylammonium chloride; VLD, vacuole-like dilation; VSV, vesicular stomatitis virus; WNK, “with no lysine” kinase; wt, wild-type.
