# Supplementary material for: Oral Antiviral Defense: Saliva- and Beverage-like Hypotonicity Dynamically Regulate Formation of Membraneless Biomolecular Condensates of Antiviral Human MxA in Oral Epithelial Cells
Source: Cells. 2024 Mar 28;13(7):590. doi: 10.3390/cells13070590 (PMC11011872; doi:10.3390/cells13070590)
Supplement: Supplementary file 1 [file cells-13-00590-s001.zip › cells-2895560-supplementary.pdf]

Supplementary Materials version 2

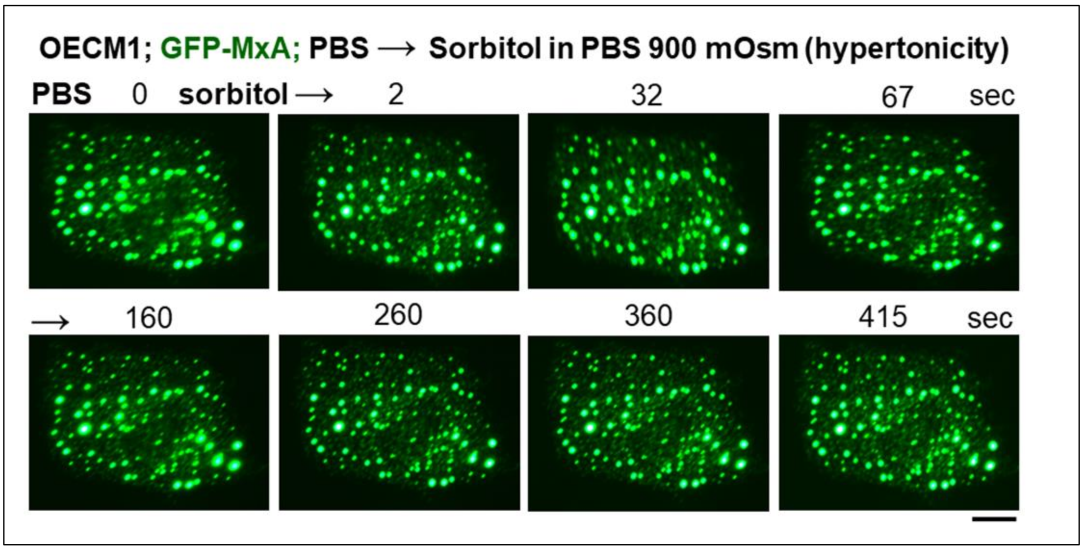

Figure S1. Lack of effect of hypertonicity (3x isotonicity) on GFP-MxA condensates in oral epithelial cells. A cell in an OECM1 culture expressing GFP-MxA condensates was first imaged in full culture medium and then shifted to culture medium supplemented with D-sorbitol (net tonicity: 900 mOsm). Live-cell imaging was carried out as indicated. Scale bar = 10 μm.

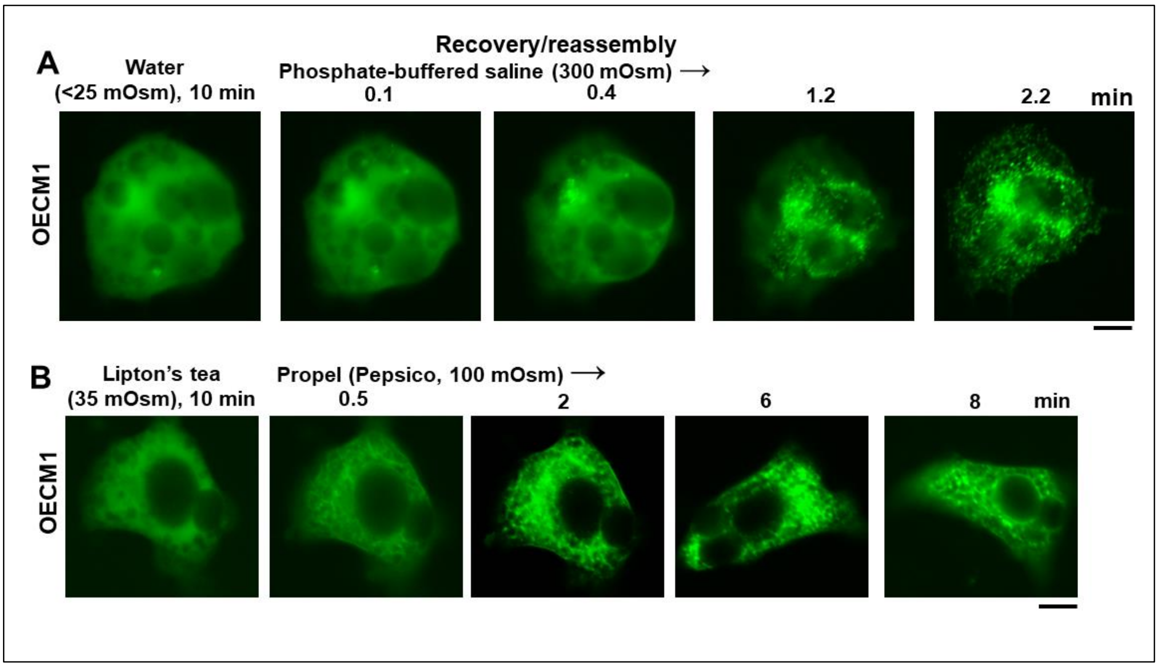

Figure S2. Panels A and B, Live-cell imaging of OECM1 cells expressing GFP-MxA condensates 2 days after transfection first observed in full culture medium, followed by exposure to water (<25 mOsm) or Lipton’s tea (35 mOsm) for 10 min to dissemble the condensates. Imaging of the same cell was continued upon shifting the cultures to phosphate-buffered saline (300 mOsm) or Propel (Pepsico)(100 mOsm). Scale bar = 10 μm

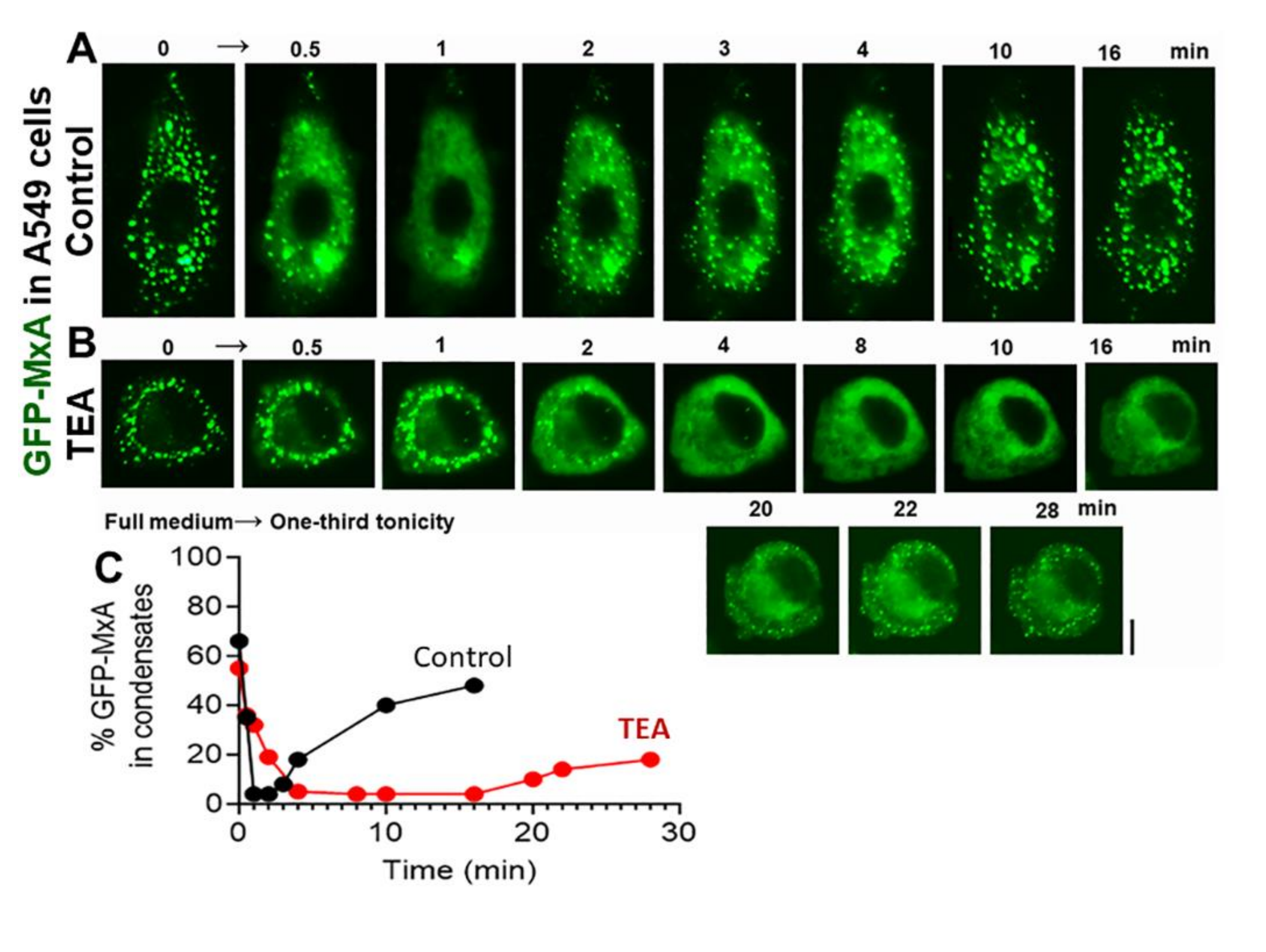

Figure S3. Testing a biochemical basis for hypotonicity sensing by GFP-MxA condensates in oral epithelial cells. Panels A and B, A549 cells were either kept in full culture medium or exposed to TEA (20 mM) in full culture medium for 20 min. Cultures were then shifted to one-third tonicity medium in the continued presence of TEA. Live-cell imaging was carried out as indicated. Scale bar = 10 μm. Panel C, Quantitation of % GFP-MxA per cell in condensates (in the same cells shown in Panels A and B).
